# Radiological Surveillance Post-Curative Colorectal Cancer Resection: Is There a Need for a Targeted Protocol?

**DOI:** 10.7759/cureus.14110

**Published:** 2021-03-25

**Authors:** Mahmoud Abdel-dayem, Lydia Maw, Edward Green, Heba Abdelaziz, P.N. Haray

**Affiliations:** 1 Colorectal Surgery, Prince Charles Hospital, Cardiff, GBR; 2 Public Health, National Hepatology and Tropical Medicine Research Institute, Cairo, EGY; 3 Colorectal Surgery, University of South Wales, Pontypridd, GBR

**Keywords:** colorectal cancer, follow up, radiological imaging

## Abstract

Background

The frequency of radiological surveillance after curative colorectal cancer resection has long been a controversial issue with the need to balance potential harm from ionizing radiation and the financial burden of intense surveillance against advantages of early detection of recurrent disease. NICE guidelines issued in 2018 suggested having two surveillance computed tomography (CT) scans within three years of surgery without specifying the timing or the interval.

Aim

To examine whether an evidence-based flexible approach based on individual patients’ risk factors can add value to surveillance protocols. Reaching a targeted protocol that can maximize early detection of metastasis without consumption of resources and most important without compromising patient safety.

Methodology

A retrospective study involving five years of data of patients who underwent curative colorectal cancer resections. Data extracted after patients completed their three-year surveillance CT scans, CT reports retrieved together with post-operative histology reports, and a detailed database was constructed.

Results

Of 179 patients included, 66 developed recurrence (7 local and 59 distant). Recurrence increased from 23.5% in T1 to 66% in T4 (P=0.0001). The median time to recurrence 23 months in T4 disease compared to 36, 42 and 43 months for stages T1, T2 and T3, respectively (P=0.0001). A similar incremental increase in recurrence noted from 22% in the N0 stage to 73.5% in the N2 stage (P=0.0001); the median time to recurrence of 14 months in N2 patients compared to 45 and 33 months for stages N0 and N1, respectively (P=0.0001). Recurrence correlated well with positive extramural vascular invasion (EMVI) status, (71.7% versus 19.3% P=0.0001) being detected significantly earlier in EMVI positive group at 17 versus 45 months (P=0.0001).

Conclusion

Flexible protocol for radiological surveillance after curative resection of colorectal cancer, based on known pathological prognostic factors, is likely to be more effective in maximizing resource utilization as well as improving patient outcomes.

## Introduction

Regular surveillance of patients following curative resection of colorectal cancer is crucial to maintain a high standard of follow-up care, ensuring early identification of any local recurrence or metastatic disease to enable further treatment to be offered. Long-standing debates around an appropriate follow-up protocol have involved, striking a balance between potential benefits of early detection of local or distant recurrence and the cost as well as potential harm to patients from ionizing radiation from multiple CT scans on the other hand.

NICE guidelines [1] issued in 2018, suggest patients should receive two CT-TAP (Computerized Tomography of Thorax, Abdomen and Pelvis) within three years following surgery. However, these guidelines do not specify the timings of these scans. A variety of follow-up protocols exist across the world but there is an urgent need for guidelines to allow for efficient and cost-effective surveillance without compromising patient safety.

Our departmental protocol, introduced in 2001, involves a clinical examination and carcino-embryonic antigen (CEA) levels every six months for five years, annual CT-TAP for three years and colonoscopy at three and six years post-operatively after all curative resections for colorectal cancer.

## Materials and methods

Aims

To examine whether an evidence-based flexible surveillance protocol, tailored to individual patients’ risk factors can be developed to maximize early detection of local or distant recurrence, without compromising patient safety.

Methodology

This was a retrospective study involving five years’ data of colorectal cancer resections (2010-2014) at Prince Charles Hospital; data were collected after the patients had completed their three annual CT-TAP, as per department protocol. The search was extended by a further year to capture any delayed investigations for a variety of reasons such as service pressures, human errors, patient delay due to unforeseen circumstances, etc.

A detailed database was constructed which included specific details of known prognostic factors in the post-operative pathology report including tumour staging (TNM classification - UICC 7th edition), extramural vascular invasion (EMVI) status and apical LN involvement as well as data from surveillance CT scans, to identify incidence and timing of any local or distant recurrences.

Eligibility criteria and selection

Inclusion Criteria

The study population was derived from adult patients who underwent colorectal cancer resections between January 2010 and December 2014 at our hospital.

Inclusion criteria were: (i) patients who had no evidence of metastatic disease at the time of surgery (stage M0), (ii) the primary treatment was performed with curative intent, (iii) the post-operative histology confirmed complete surgical resection (R0), (iv) patients who were deemed fit enough to undergo follow-up and engaged in the follow-up protocol either until the completion of annual CT-TAP for three years or until they were diagnosed with a local or distant recurrence.

The main outcome measures were the primary outcome was the identification of local or distant recurrence.

Statistical techniques

Data analysis was carried out using IBM SPSS® (version 24, IBM Corp., Armonk, NY). Identification of any correlations between known prognostic risk factors and recurrences was carried out using Kaplan Meir and Chi-square tests and a P-value of <0.05 was considered significant.

## Results

Due to the inclusion criteria, only 179 patients were included in the study. There were 103 males and 76 females (M:F = 1.35:1) with a median age of 63 (range 30-96) years.

The operative procedures performed included 57 total mesorectal excisions, 29 left-sided segmental resections, 78 right-sided segmental resections and 15 multi-segmental resections. The operations were performed by three colorectal surgeons in the department. Out of the 179 procedures, 18 were performed as open approach (10%) and 161 as a laparoscopic approach.

Out of the 179 patients, 66 (36.9 %) developed recurrence, of which 7 were local and 59 were distant recurrences. Correlations with known poor prognostic indicators confirmed the following findings.

T-stage

**Figure 1:**
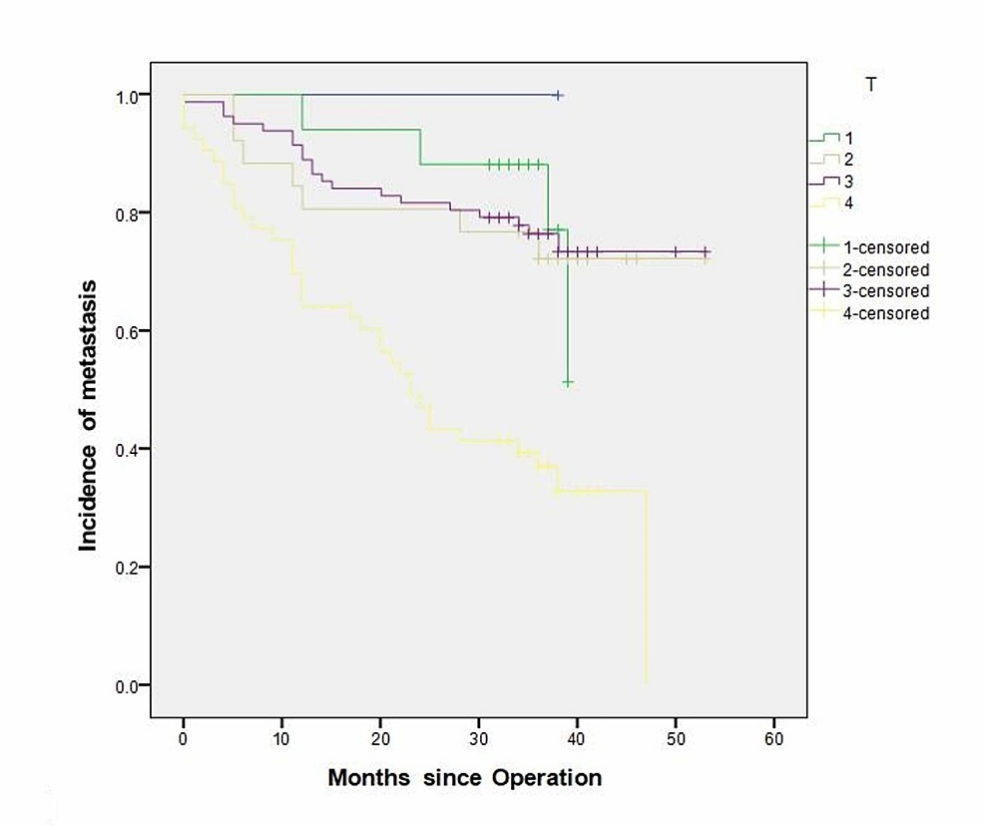
Incidence of recurrence in relation to T-stage

The incidence of recurrence increased from 4 out of 17 (23.5%) in T1, one of which was a local recurrence, 7 out of 26 (26%) in T2 (one local), 20 out of 82 (24.4%) in T3 (one local) and 35 out of 53 (66%) in T4 (4 local recurrences). The incidence of recurrence in T4 tumours was found to be higher than any of the other T stages, achieving statistical significance (P=0.0001). The median time to recurrence was 23 months after the operation for patients with T4 disease (CI 95% “17.905-28.95”), compared to 36, 42 and 43 months for stages T1, T2 and T3, respectively. This earlier recurrence in patients with T4 tumours is statistically significant (P= 0.0001; Figure 1).

N-stage

**Figure 2:**
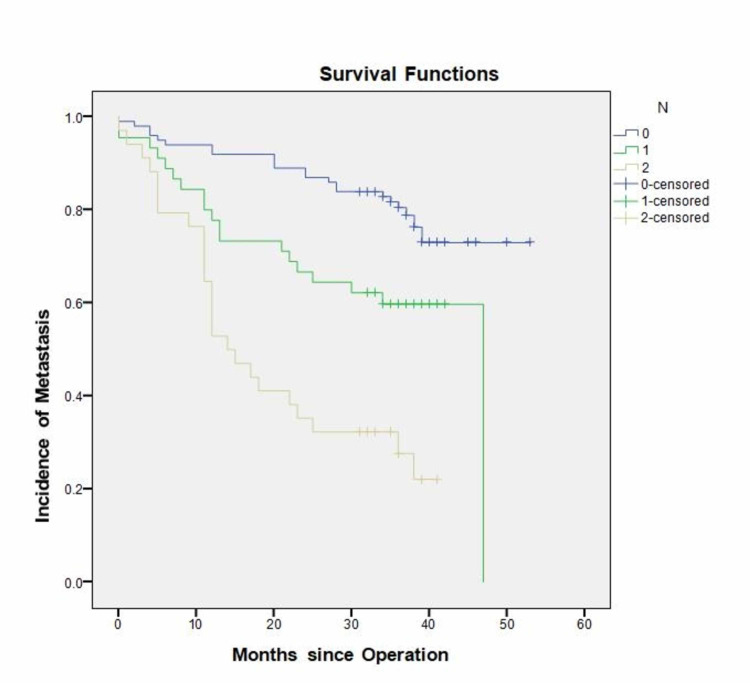
Incidence of recurrence in relation to N-stage

The incidence of recurrence increased from 22 out of 100 (22%) in the N0 stage (3 local) to 19 out of 45 patients (42.2%) in the N1 stage (2 local) and 25 out of 34 patients (73.5%) in N2 stage, 2 of which were local recurrences. Patients with stage N2 disease were at a significantly higher risk of recurrence (P = 0.0001) when compared to patients with N0 and N1 disease. The median time to the occurrence was also significantly earlier (P=0.0001) at 14 months (CI 95% “9.102-18.898”) after the operation for patients with N2 disease, compared to 45 and 33 months for stages N0 and N1, respectively (Figure 2).

EMVI

**Figure 3:**
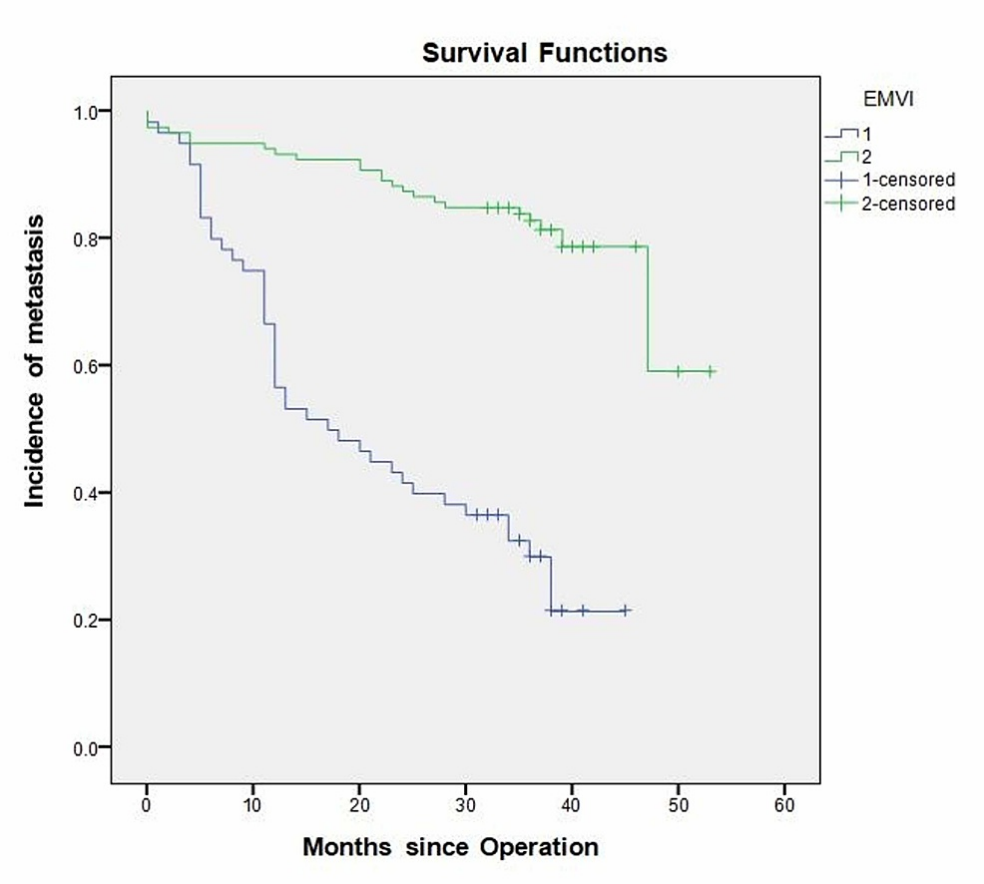
Incidence of recurrence in relation to extramural vascular invasion status

Sixty patients out of the 179 were EMVI positive (33.5%). Recurrence occurred in 43 out of these 60 patients (71.7%), compared with 23 out of 119 (19.3%) in the EMVI negative group. This difference was statistically significant (P = 0.00001). Recurrence was also seen to occur significantly earlier in the EMVI positive group at 17 months (CI 95% 7.24-26.76) versus 45 months (CI 95% 41.74-48.28) in the EMVI negative group (P = 0.0001; Figure 3).

Apical lymph node

**Figure 4:**
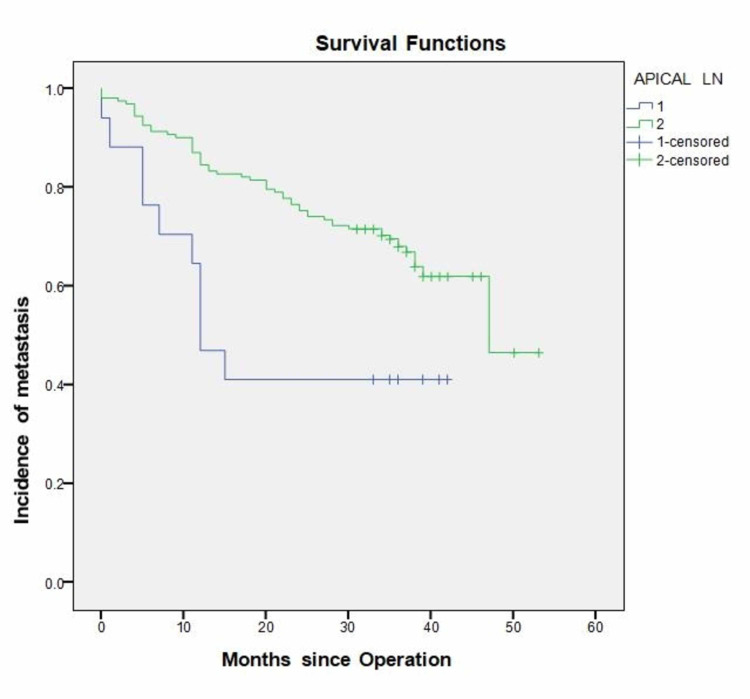
Incidence of recurrence in relation to apical lymph node involvement status

In our practice, we routinely mark the highest lymph node (LN) resected (the closest to the root of mesentery) and mark it with a stitch for the pathologist. This LN is clearly reported in the pathology report as apical LN. Apical LN tumour infiltration was noted in 17 (9.5%) patients. Of these, 10 (58.8%) went on to develop recurrence compared with 56 out of the 162 apical LN negative patients (34.6%). This difference reached statistical significance (P = 0.049). Time to recurrence in the apical LN positive group was also significantly earlier (P = 0.006) at 12 months (CI 95% “7.966-16.034”) compared with 39 months (CI 95% “36.166-42.504”) in the apical LN negative group (Figure 4).

There was no statistically significant difference in the incidence or timing of recurrence in relation to basic demographic data (patient sex or age). T4 tumour stage, N2 disease, apical LN involvement and EMVI positivity were all independent predictive factors, not only of recurrence but also of early recurrence; however, due to the small sample size, a multi-factorial analysis did not yield meaningful results.

## Discussion

Our study has demonstrated a high risk of recurrence in T4 tumours, N2 stage, EMVI and apical LN involvement, and these findings are in broad agreement with many other publications [2-5]. Though there have been many RCT comparing various follow-up protocols, all of them have taken a standard approach to all operated cases rather than trying to identify a more tailored approach based on specific prognostic factors.

The follow-up after colorectal surgery (FACS) trial [6], a randomized controlled trial to assess the cost-effectiveness of intensive versus scheduled follow-up in patients who have undergone resection for colorectal cancer with curative intent, concluded intensive imaging or CEA screening each provided an increased rate of identification of recurrence (treatable by surgery with curative intent), compared with minimal follow-up. However, the absolute difference in curative resection of recurrence measured only 5%, and the number of deaths did not differ between the more intensive monitoring cohorts and the minimum follow-up cohort.

A large series (8529 cases) published from the USA [7], showed similar results with no significant differences in rates of resection for cancer recurrence or overall survival identified between hospitals offering high-intensity CT or CEA surveillance versus those with low intensity follow up. Surveillance testing has obvious benefits, but the optimal frequency of testing remains in question. The National Institute for Health and Care Excellence (NICE) in the UK [8] has issued guidelines in 2018 advocating CEA testing every six months and two CT-TAP during the first three years after curative surgery, based on recent evidence from the FACS, CEA watch, GILDA and COLOFOL trials [6,9-11]. The timing of these surveillance scans was, however, left unclear. Once again, this is guidance that is meant to apply to all cases and not based on any specific prognostic criteria.

We believe that the reason why so many trials have not shown any significant advantages of intensive follow-up is that they have included all cases rather than grouping patients by their prognostic features. Our study, presented here, has focused on finding a more tailored approach based on the post-operative histopathological features. We have been able to demonstrate that the post-operative histological tumour staging, the presence or absence of EMVI and apical LN involvement can be used as guides to be able to categorize patients into low-intensity or high-intensity surveillance cohorts. We feel that such a more structured approach for follow-up of patients after curative colorectal cancer surgery is necessary, not only to improve early detection of treatable recurrence but also to maximize effective utilization of resources. Further studies are needed to explore this concept.

## Conclusions

This study has demonstrated that having a more targeted approach with the intensive follow-up being offered to patients who are at higher risk of recurrence. Patients with post-operative histopathological adverse prognostic factors including T stage 4, N stage 2, involvement of apical LN and EMVI positive have higher incidence together with early occurrence of metastasis and targeted, more intense approach to this cohort will lead to earlier detection of recurrences while maximizing resource utilization.
